# Geriatric Oncology: From Research to Clinical Practice

**DOI:** 10.3390/cancers13225720

**Published:** 2021-11-15

**Authors:** Nienke A. de Glas

**Affiliations:** Department of Medical Oncology, Leiden University Medical Center, Albinusdreef 2, 2300 RC Leiden, The Netherlands; n.a.de_glas@lumc.nl

The incidence of cancer in older adults is strongly increasing due to the ageing of the population [1]. In recent years, the treatment of older adults with cancer has received much attention, and research relating to Geriatric Oncology has developed considerably. This is represented in this Geriatric Oncology Special Issue, which includes 14 original research papers and 2 reviews.

In order to improve individualized decision making in older adults with cancer, predictive tools can be used in daily clinical practice [2]. Several papers in this Special Issue focused on the prediction of outcomes in older adults with cancer. Kirkelund Bentsen et al. showed that the combination of G8 with handgrip strength improved the prediction of overall survival in patients with non-small cell lung cancer [3]. Similarly, Feliu et al. showed that geriatric assessment can predict unplanned hospitalizations in patients undergoing chemotherapy [4]. The study by de Boer et al. showed that the number of comorbidities was highly predictive of overall survival in patients with early-stage breast cancer [5].

Another way to further improve the prediction of outcomes in older adults with cancer is to study predictive biomarkers. The study by Di Donato and colleagues showed that metabolomics are highly predictive of prognosis in older adults with metastatic colorectal cancer [6]. Berben et al. studied the predictive capacity of immune senescence markers in older patients with breast cancer and found an association between an senescent immune profile and clinical markers of frailty [7]. In addition, Berben et al. provided a review in which the connection between cellular senescence in ageing and cancer was summarized [8].

Furthermore, several studies investigated the use of predictive models in different settings. Souwer et al. developed a prediction model to predict postoperative complications in older adults with early-stage colorectal cancer [9]. Additionally, in the operative setting, Ferroli and colleagues developed a predictive model for outcomes after brain surgery in older adults, and showed that the main predictor was the occurrence of postoperative complications [10].

Another important aspect in improving outcomes in older patients with cancer is the organization of health care systems. Soto-de-Perez-Calis and colleagues validated a Spanish version of the Geriatric assessment in order to improve health care for older Spanish-speaking patients [11]. The review of Filteau and colleagues provided an overview of cultural and ethical barriers in treating nursing home residents with cancer [12]. Additionally, the study of DuMontier and colleagues investigated the use of electronic medical records to measure frailty status in veterans [13].

Finally, several studies described the outcomes of older patients with cancer in different settings. De Glas et al. showed that older patients with metastatic melanoma who were treated with immunotherapy had similar chances of response and toxicity compared to younger trial populations [14]. Couderc and colleagues investigated the outcomes of older men with metastatic prostate cancer, and showed that cardiovascular events increased with increasing age but were not associated with a worse overall survival [15]. Liposits and colleagues showed that upfront dose-reduced chemotherapy in the NORDIC9 randomized trial resulted in a better quality of life and physical functioning compared to full-dose chemotherapy in patients with metastasized colorectal cancer [16]. Additionally, the study by Okano investigated differences in outcomes between younger and older patients with breast cancer, and showed that octogenarians had unfavorable tumor micro environment profiles and a worse survival [17].

In conclusion, this Special Issue included a broad range of papers enhancing the field of Geriatric Oncology, mainly aiming at further individualizing the treatment of older adults with cancer. As the population of older adults with cancer will continue to rise, these types of studies are important to address the specific needs of this heterogeneous population. We would, therefore, like to thank all participating authors and reviewers for their contribution to this Geriatric Oncology Special Issue.
